# Partisan Influence on Policy Preferences in Retrenching the Welfare State

**DOI:** 10.1093/poq/nfaf017

**Published:** 2025-05-29

**Authors:** Miroslav Nemčok, Hanna Wass, Juho Vesa

**Affiliations:** Postdoctoral Fellow, Department of Political Science, University of Oslo, Oslo, Norway; Adjunct Professor and Vice-Dean, Faculty of Social Science, University of Helsinki, Helsinki, Finland; Academy Research Fellow, Faculty of Social Science, University of Helsinki, Helsinki, Finland

## Abstract

While citizens typically favor welfare policies, the electoral consequences of retrenching the welfare state are often minimal for parties implementing the reforms. Using two structural reforms in Finland as a natural quasi-experiment, we show that voters’ policy preferences shift in response to welfare reform measures initiated by their preferred parties. In December 2020, the Finnish center-left government enacted two reforms: one reducing social protection by removing entitlements for laid-off older workers to receive income-based unemployment benefits, and the other increasing social spending by extending the compulsory education age from 16 to 18. Using a two-wave panel survey conducted before and after the government actions, the results indicate that government voters became considerably more supportive of both reforms, despite their initial low support for welfare retrenchment and its contradiction with the established ideological profile of their parties. Moreover, the shift in voters’ policy preferences was substantively greater compared to their opposition counterparts and not affected by ideology and economic self-interest. Hence, voters’ policy preferences show dynamic adaptability to match the party line, thereby reducing grounds for holding the parties accountable.

## Introduction

When political parties aim at retrenching the welfare state by reducing the size or scope of social protection, their voters can respond in one of two ways. According to existing literature on welfare attitudes, voters’ support for welfare state policies is remarkably stable and high (Brooks and Manza 2006, 2007), which suggests that they may seek avenues to hold parties accountable for conducting such unpopular actions (Pierson 1996, 2001). The literature on partisan cues, in turn, suggests a different dynamic. Here, voters are expected to follow their preferred party’s lead in navigating their way through the complex policy space and flexibly update their policy preferences so they are better aligned with the party line (Peterson 2019; Slothuus and Bisgaard 2021b).

Two structural welfare reforms enacted in Finland around the same time provide a suitable context to empirically examine both theories simultaneously. In December 2020, the Finnish center-left government initiated a *welfare retrenchment* reform that removed the entitlement of laid-off older workers to receive income-based unemployment benefits before reaching the age of retirement (also known as the “retirement tube”). Having been on the political agenda for decades, the decision was described as historical for contradicting the preferences of left-wing voters, who constituted a substantial support base for the center-left government. Only a few days before, the parliament had approved a government bill to extend compulsory schooling age from 16 to 18 and provide school supplies free of charge. This *welfare expansion* reform, categorized as a social investment (Garritzmann 2021), increases public spending, and hence, it denotes a progressive approach to welfare.

Using an original two-wave panel survey conducted among the same respondents before (in September 2020, n = 2,106) and after (in February 2021, n = 1,701) the enactment of the two policies, we find that the partisan cues provided by the center-left government parties increased voters’ support for both reforms. The magnitude of opinion change regarding welfare retrenchment, initially opposed by most government voters, was comparable to that observed for the welfare expansion reform, which initially enjoyed broader support among government voters. Moreover, both preference shifts were substantially larger than those observed among opposition voters. The results remain consistent across various empirical tests, including an improved causal identification strategy using individual fixed effects (i.e., first-difference) models.

All told, government voters substantially increased their support for the retrenchment policy, despite its inconsistency with their parties’ ideological profiles. Additional subgroup analyses show that partisan cues influenced voters similarly regardless of their own ideological position on the left-right political spectrum and the economic incentives associated with the specific reforms. The findings suggest that voters have a readiness to align their policy preferences with the party’s stances, even when it contradicts their own ideological orientation and self-interest considerations. Such elasticity also reduces voters’ motivation to hold parties accountable in the next elections. This finding suggests one explanation for why stringent research designs and extensive comparative studies have struggled to identify electoral penalties targeting the parties responsible for welfare state retrenchments (Armingeon and Giger 2008; Giger 2011; Ahrens and Bandau 2023). If voter preferences are elastic, leading them to adopt even ideologically inconsistent policy stances, the electoral punishment becomes unnecessary.

In using a natural quasi-experimental design, we deepen existing scholarly understanding of the development of voters’ policy preferences in real-world settings. Building on the findings presented by Slothuus and Bisgaard (2021b), who focused on welfare retrenchment initiated by center-right and right-wing parties, we demonstrate that voters are willing to adopt ideologically inconsistent stances even when center-left governments introduce a retrenchment policy. Furthermore, we provide a systematic theorization of how partisan cues bring elasticity into voters’ policy preferences, even in the context of welfare state retrenchment. Previous studies suggest that voters’ policy preferences are generally stable in favor of established levels of social protection (Brooks and Manza 2006, 2007) and induce negative sentiments toward parties implementing retrenchment reforms (Giger 2011, 2012; Schwander and Manow 2016; Arndt, Jensen, and Wenzelburger 2021). However, our empirical analysis demonstrates that many voters adjust their policy preferences to match the party line when their preferred party decides to retrench the welfare state. In fact, we show that voters are responsive to party cues even when the party’s policies contradict voters’ political ideologies as well as when the reform reduces social protection with direct implications for their personal well-being.

Our study therefore complements earlier findings by Nemčok, Wass, and Vesa (2024), which indicated that strongly attached partisans are more responsive to party cues in the case of unpopular policy reform compared to weakly attached partisans, who adjust their policy preferences later. In addition to that study, our findings show that voters respond to party cues irrespective of their self-interest and political ideologies (i.e., self-placement on the left-right spectrum). Hence, these findings suggest that party cues reduce the grounds for holding parties accountable for welfare state retrenchment. Importantly, it is not that voters cannot hold parties accountable; rather, they choose not to. This provides an important insight, improving our understanding of the formation of voters’ policy preferences in a real-world context.

### Stable Welfare State Preferences Among Voters

Voters’ preferences for welfare state policies are influenced by the nuanced character of redistribution dynamics, which are in turn motivated by self-interest considerations (Kangas 1997), context-dependent cultural understandings of welfare (Jo 2011), and perceptions of social justice (Van Hootegem, Abts, and Meuleman 2020). Generally speaking, voters favor policies involving more “redistribution to,” positioning them among likely recipients of welfare support, compared to less favored “redistribution from” policies, which emphasize a contributory dimension rooted in social solidarity (Blekesaune and Quadagno 2003; Cavaillé 2023).

In theoretical accounts of welfare preferences, citizens are assumed to be informed, self-interested maximizers (Meltzer and Richard 1981) who prefer social policies that prevent their living standards from declining (Hacker, Rehm, and Schlesinger 2013; Owens and Pedulla 2014). While in rare instances voters’ preferences may reflect their own experience with social protection (Margalit 2013; Naumann, Buss, and Bähr 2016), the complexity of modern welfare states makes it difficult to assess the redistributive implications of social policies for everyday living conditions (Giger 2011; Cavaillé and Trump 2015). In most cases, voters’ preferences for welfare policies are rooted in two *fairness* norms relevant to redistribution: the *proportionality* principle, which follows market logic suggesting that social benefits should be allocated based on contribution and merit rather than on need alone, and the *reciprocity* principle, which underpins social solidarity and mutual aid (Cavaillé 2023). Additionally, the conception of social solidarity tends to prevail when redistribution targets deserving groups (e.g., the elderly, sick, disabled), in line with the CARIN (Control, Attitude, Reciprocity, Identity, and Need) principles (Meuleman, Roosma, and Abts 2020), rather than those perceived as responsible for their own circumstances (e.g., unemployed) or those lacking in reciprocity as welfare recipients (e.g., immigrants) (Applebaum 2001; van Oorschot 2006; Jensen and Petersen 2017).

When welfare policy preferences are assessed through the lens of fairness norms, they often become less influenced by momentary self-interest and instead develop into more stable attitudes that more closely resemble values (Jo 2011; Van Hootegem, Abts, and Meuleman 2020). While such preferences can be adjusted, it requires an exceptionally strong impulse and suitable contextual conditions (Marx and Schumacher 2016; Naumann 2017). Otherwise, voters’ welfare state preferences remain stable even amid welfare state changes (Armingeon and Giger 2008; Achterberg, van der Veen, and Raven 2014; Ahrens and Bandau 2023) and increasing income inequality (Alesina and Glaeser 2004; Cavaillé 2023).

Such high and stable public support for welfare policies (Brooks and Manza 2006, 2007) should pose a risk of electoral backlash for any party reducing the level of social protection (Pierson 1996, 2001). While a few case studies suggest that welfare retrenchment may have negative electoral consequences (Schwander and Manow 2016; Arndt, Jensen, and Wenzelburger 2021), large comparative analyses fall short of generalizing such a pattern (Armingeon and Giger 2008; Ahrens and Bandau 2023). At best, electoral punishment applies to pro-welfare state parties (Giger and Nelson 2013; Schumacher, Vis, and van Kersbergen 2013; Horn 2021; Langsæther, Goubin, and Haugsgjerd 2022), particularly in contexts where it is clear to voters which party is responsible for making the cuts (Giger 2011, pp. 48–51), even when fiscal austerity measures are arguably a reasonable response to mounting public debt (Hübscher, Sattler, and Wagner 2021). Moreover, the effect appears only in the case of a sizable reduction in social protection (Armingeon and Giger 2008), with the most pronounced negative influence observed among voters who are especially interested in the topic (Giger 2011, 2012).

### Partisan Cues Add Elasticity in Voters’ Policy Preferences

While parties looking for electoral victories should be discouraged from retrenching the welfare state, such reforms are in fact quite common (Allan and Scruggs 2004; Bridgen 2019) and seldom meet with severe punishments from voters (Armingeon and Giger 2008; Giger 2011, 2012; Schumacher, Vis, and van Kersbergen 2013; Ahrens and Bandau 2023). One potential reason for this puzzling discrepancy lies in the character of policy preferences (for a review of other explanations, see Ahrens and Bandau 2023). The literature on the electoral consequences of welfare retrenchment often perceives voters’ policy preferences as a steady component that rarely forms favorable conditions for reforming the welfare state without the fear of electoral backlash for governing parties (Giger and Nelson 2011, 2013).

In contrast, the literature on partisan cues generally argues that voters’ preferences are elastic, suggesting that voters often follow their favored party and respond to its actions by adjusting their own preferences accordingly (Broockman and Butler 2017; Barber and Pope 2019; Slothuus and Bisgaard 2021b; Nemčok, Wass, and Vesa 2024). This pattern holds true in a variety of contexts and for a range of policy issues (Satherley et al. 2018; Nordø 2021; Schonfeld and Winter-Levy 2021; Slothuus and Bisgaard 2021a), although there are some limits to partisan loyalty (see Mullinix 2016; Mummolo, Peterson, and Westwood 2021).

Such adaptation is possible because party attachment constitutes an integral part of voters’ social identities (Greene 1999; Green, Palmquist, and Schickler 2002; Leeper and Slothuus 2014). Voter partisanship establishes a psychological predisposition or *perceptual screen* through which individuals perceive and interpret social reality (Gaines et al. 2007; Jerit and Barabas 2012), thereby shaping the formation of their political preferences and behavior (Nemčok, Spáč, and Voda 2019; Slothuus and Bisgaard 2021a). This process is associated with party supporters’ tendency to engage in *motivated reasoning*, which presupposes that they change their opinion to remain aligned with the party, often adopting party-provided justifications for preference shifts (Petersen et al. 2013; Bolsen et al. 2014; Leeper and Slothuus 2014). Therefore, partisan attachment brings ideological flexibility to the issue preferences of voters precisely because it is considerably easier to adjust one’s opinion on a single policy issue than to replace a profound and often habitual party attachment with a similarly complex connection to another party (Petersen et al. 2013; Chong and Mullinix 2019).

The capability for elasticity becomes especially apparent in the context of social policy reforms that seem distant from voters’ immediate concerns, rendering them low-stake issues with consequences that are difficult to assess beforehand (Cavaillé 2023). In a multifaceted and information-rich environment, most voters rely on party cues to navigate the multifold political space (Bisgaard 2015) rather than engaging in the cognitively demanding task of understanding the policy implications on their own (Sniderman and Stiglitz 2012; Bisgaard and Slothuus 2018). Following this logic, welfare state reform should constitute a suitable environment in which voters dynamically update their preferences to align with the party line.

### The Empirical Context: Two Structural Welfare Reforms in Finland

The two above-discussed theories on welfare attitudes and partisan cues offer contrasting expectations regarding voters’ responses to welfare state retrenchment initiated by their preferred parties. Two adjustments to the welfare system in Finland that occurred in December 2020 provide a suitable context for empirically examining voters’ reactions vis-à-vis their theoretical expectations. Halfway through its term in office and against the backdrop of the COVID-19 pandemic, the Finnish center-left government enacted two welfare reforms that had been on the political agenda for more than two decades.^1^ Notably, neither the government nor any other relevant stakeholder explicitly associated the reforms with the pandemic or justified them on such grounds.

First, on December 17, 2020, the government parties unanimously decided to gradually phase out the unemployment pension, the so-called “retirement tube,” beginning in 2023. As a result of this *welfare retrenchment* reform, individuals over 55 years of age who were laid off from their jobs were no longer eligible for an earnings-related unemployment allowance before reaching retirement age. The government’s intention was to disincentivize early labor market exits, and hence, to increase the employment rate. Although the reform theoretically applies to an entire cohort, its primary aim was to eliminate “redistribution to” those who were pushed from the employers’ side into early retirement without a considerable worsening in earnings. This change is closely tied to increased fairness of the system by making retirement benefit conditions more proportional for all.

The second enacted reform increased social spending by extending the compulsory school age from 16 to 18 years, accompanied by a decision to provide secondary-level study material free of charge. It was initiated by the government on October 15 and approved by the parliament on December 15, 2020. This *welfare expansion* reform shared the goal of enhancing the employment rate, albeit through considerably different means: increasing the contributory side of the working population by “redistributing to” young people, who generally do not score particularly high in terms of their deservingness status (Reeskens and van der Meer 2017; Heuer and Zimmermann 2020). The aim was to improve their knowledge and the skills required in labor markets, thereby reducing the likelihood of unemployment and the need for economic support.

While government parties backed both reforms unanimously, the largest opposition parties were split on the “retirement tube” removal measure: the right-wing populist Finns Party opposed it, while the traditional right-wing National Coalition Party supported it. All opposition parties opposed extending compulsory schooling age. Importantly, such oppositional stances remained consistent between the survey rounds.

The reforms vary considerably in terms of their impact on the welfare system, altering the status of beneficiary groups and the fairness of redistribution. Their initial popularity differed among party faithful, with the removal of the “retirement tube” being considerably less popular than the extension of compulsory schooling (see Supplementary Material section B). This makes the reforms an excellent context for studying voters’ responses to welfare reforms.

## Data and Methods

The analysis uses a two-wave panel survey, which involved approaching the same respondents twice. The respondents for the first wave were selected through stratified sampling from an actively managed internet panel of approximately 40,000 adult Finnish citizens administered by the survey agency *Taloustutkimus*. Quotas based on gender, age group, and residence (NUTS 2) were applied to ensure representativeness of the adult Finnish population.

The first wave took place on September 1–3, 2020, more than three months before the Finnish government’s initiative to abolish the retirement tube and the parliament’s approval of the schooling reform. One invitation to participate in the survey, followed by one reminder, was issued to approximately one-quarter of the whole internet panel (n = 11,479). In the first wave, 2,106 respondents completed the CAWI questionnaire, resulting in an 18.3 percent response rate.

The second wave was conducted between February 12 and 17, 2021, approximately two months after the reforms had been initiated by the government. All participants from the first wave were invited to participate, with one reminder issued after the initial invitation. In total, 1,701 respondents completed the questionnaire, resulting in a (return) response rate of 80.1 percent.

The analysis is solely based on the data for respondents who participated in both waves. After accounting for listwise deletion of missing data—primarily due to missing party and policy preferences—and limiting the sample to those with consistent party preferences across waves,^2^ in addition to excluding nonvoters, the available unique respondents for analysis ranged from 944 to 1,108.

Members of the *Taloustutkimus* internet panel are Finnish adults recruited through both offline and online methods. Offline recruitment includes national representative telephone and face-to-face (door-to-door) surveys, as well as other campaigns involving telephone or mail outreach, all using random sampling. Online recruitment leverages a range of digital channels, including direct emails, network media advertisements, and Facebook. The panel is actively managed to ensure quality by monitoring quality of responses, response rates, and activity levels, with inactive members being removed regularly.

The main focus of this study lies in assessing voters’ *policy preferences*, which are embedded within a battery of policy-related questions, as shown in table 1. In the first wave, the questionnaire began with an extensive list of policy proposals, without any indication of whether or not the government would pursue them. This was followed by questions asking respondents to self-assess their social status, occupation, and income, both in comparison to others and in relation to their own past experiences. Since respondents’ socio-economic background (gender, age, education, occupation, residence, and household) was already collected during panel enrollment, these demographic questions were not repeated in either wave of the survey.

**Table 1.** Operationalizations of policy preferences.

Shared introduction:	
The COVID-19 pandemic has driven societies into a period of crisis and made it more difficult to find solutions to many already prevailing social problems. In this survey, we study citizens’ support for different policy solutions aimed at enhancing the economy and employment.	

First round	Second round
What do you think about the following policy proposals?	The Finnish Parliament has recently approved the following reforms. What do you think about this decision?

*Abolish the “retirement tube,” which allows elderly individuals who are unemployed to receive income-based unemployment compensation until reaching retirement age.*	*Abolish the “retirement tube,” which allows elderly individuals who are unemployed to receive income-based unemployment compensation until reaching retirement age.*
Unemployment benefits will be structured in tiers, initially providing a larger benefit than currently, but gradually decreasing as unemployment persists, and being paid for a shorter duration.	
Changes will be made to unemployment benefits so that the earnings-related daily allowance will no longer contribute to pension benefits in the future.	
The system of partial unemployment benefits, which provides compensation for reduced working hours, will be phased out.	
Wage increases will be implemented at a slower rate compared to competitor countries, making it more advantageous for employers to hire workers.	
Working hours will be extended or adjusted to reduce overtime costs for employers.	
The practice of negotiating terms of employment and wages locally within companies will be expanded.	
A transition to a six-hour workday will be pursued.	
Adult education support will be discontinued, leaving study grants or the option to study with unemployment benefits as the primary means of support for adult education, especially for courses that promote employment.	
Monthly credit requirements for study points will be strengthened to accelerate progress in studies.	
*Extend the compulsory schooling age to 18 years.*	*Extend the compulsory schooling age to 18 years.*
Ensure that secondary education is fully free of charge, covering not only tuition but also study materials and tools.	Ensure that secondary education is fully free of charge, covering not only tuition but also study materials and tools.

Shared set of responses offered to each question in both rounds:	

Strongly agree	Strongly agree
Agree	Agree
Disagree	Disagree
Strongly disagree	Strongly disagree
Don’t know	Don’t know

*Note:* The question wording in the second round was intentionally designed to inform the respondents about the current status of the reforms. Not mentioning that the reforms had already been enacted would have hindered our ability to reliably determine which respondents were aware of the reforms, used as a “treatment” in the research design.

The survey batteries included more questions; the two questions used in this study are italicized.

In the second round, the questionnaire began by asking respondents three key questions on policy preferences, while the remaining items from the original battery were omitted to optimize resources.^3^ Additionally, the second round included questions on political attitudes (e.g., issue saliency, party attachment, political interest), which have not been used due to a concern about posttreatment bias. The survey was conducted independently, with no additional questions asked concurrently.

On average, respondents took approximately 9 minutes to complete the first round of the survey, while the second wave required only about 5 minutes due to a shorter questionnaire. Participants did not receive additional financial or other forms of compensation for taking part in this survey. Given that *Taloustutkimus* panel members are surveyed frequently—on average, twice a week^4^—they are unlikely to recall being asked similar questions about the reforms within a six-month period, thereby reducing the risk of consistency bias—a common concern in panel designs with repeated individual-level measurements.

## Results: Parties’ Preference-Shaping Potential

**Figure 1.**
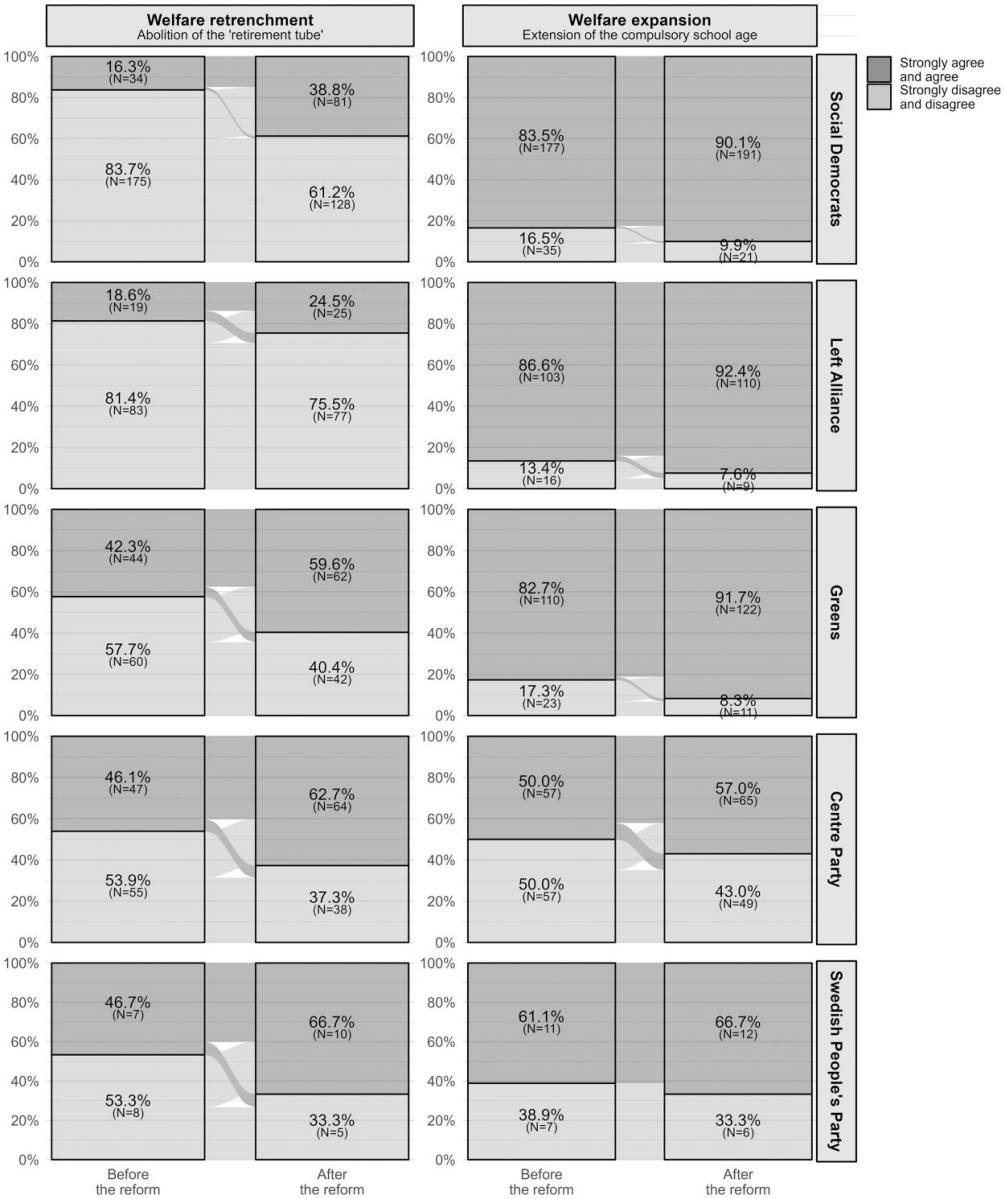
Changes in the policy preferences of government party voters. The figure combines respondents who strongly agree and agree, as well as those who strongly disagree and disagree. The distribution of these groups is shown within the electorates of individual government parties.

We first descriptively examine the changes in policy preferences. Figure 1 displays the policy stances of the government voters before and after enactment of the policies.^5^ Notably, support for welfare retrenchment surged by 17 percentage points, to 22 percentage points (depending on the party), across all government party electorates, with the exception of the Left Alliance, where the increase was smaller, at six percentage points. This increase contrasts with opposition party voters, where only supporters of the Christian Democrats showed a comparable increase in support (see Supplementary Material section C). In the case of welfare expansion, while government supporters revealed a modest increase in support from five to nine percentage points, opposition voters revealed mostly a decrease of comparable magnitude. The trends underscore that government voters were more prone to increase their support for both welfare reforms.

The substantial similarity in the responses of both government and opposition voters to welfare reforms allows us to streamline our subsequent analysis by focusing on the two groups. Table 2 presents the results of fixed effects (first-difference) models examining within-individual effects, revealing that policy enactments had a significantly larger effect on government voters.^6^ Their support for welfare retrenchment increased by 13.3 percent (or 0.4 points on the provided four-point scale), compared to a 4.3 percent effect among opposition voters. Regarding welfare expansion, government supporters exhibit an 8.7 percent increase, whereas no discernible shift is evident among opposition voters. Importantly, the results remain robust even after accounting for potential time-variant ideological shifts (see models 2 and 4).

**Table 2.** Within-individual shifts in policy opinions among government and opposition voters.

	Welfare retrenchment: Abolition of the “retirement tube”						Welfare expansion: Extension of the compulsory schooling age					
	(1)			(2)			(3)			(4)		
After the reform (February 2021)	0.129	[0.043]	(0.002)	0.130	[0.043]	(0.002)	−0.025	[0.040]	(.521)	−0.028	[0.040]	(0.489)
After the reform * government voters	0.281	[0.057]	(0.000)	0.280	[0.057]	(0.000)	0.259	[0.049]	(.000)	0.259	[0.049]	(0.000)
Left-right self-placement				−0.011	[0.027]	(0.688)				0.023	[0.025]	(0.359)

Individual fixed effects	Yes			Yes			Yes			Yes		
S.E.: clustered	by individuals			by individuals			by individuals			by individuals		
Observations (unique individuals)	2,008 (1,004)			2,008 (1,004)			2,216 (1,108)			2,216 (1,108)		
R^2^	0.808			0.808			0.866			0.866		
Within R^2^	0.106			0.106			0.044			0.045		

*Note*: Fixed effects (first-difference) models are estimated. Coefficients are unstandardized OLS regression estimates with individual cluster-robust standard errors in brackets and *p*-values in parentheses.

**Figure 2.**
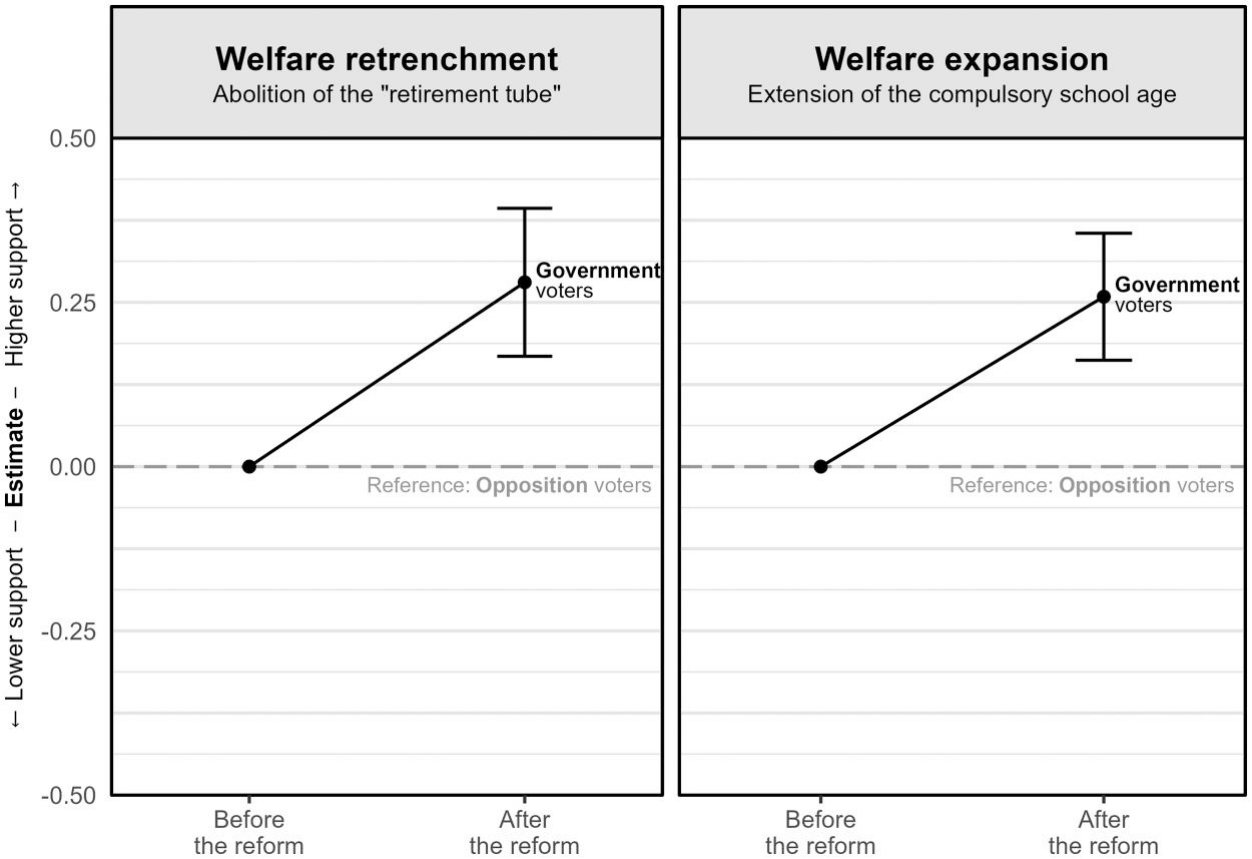
Within-individual shifts in policy opinions among government voters. Attitudes among opposition voters are used as the reference group. The coefficients are derived from table 2. Vertical lines represent 95 percent confidence intervals based on individual cluster-robust standard errors.

Given that all voters may be affected by similar unobserved factors, figure 2 illustrates the gap in policy preferences between government and opposition supporters. This gap, representing the interaction term in models 1 and 3 of table 2, highlights the similarity between changes in policy preferences among government voters: their policy preferences improved by 0.28 (welfare retrenchment) and 0.26 points (welfare expansion), when compared to the opposition voters as a reference.

**Figure 3.**
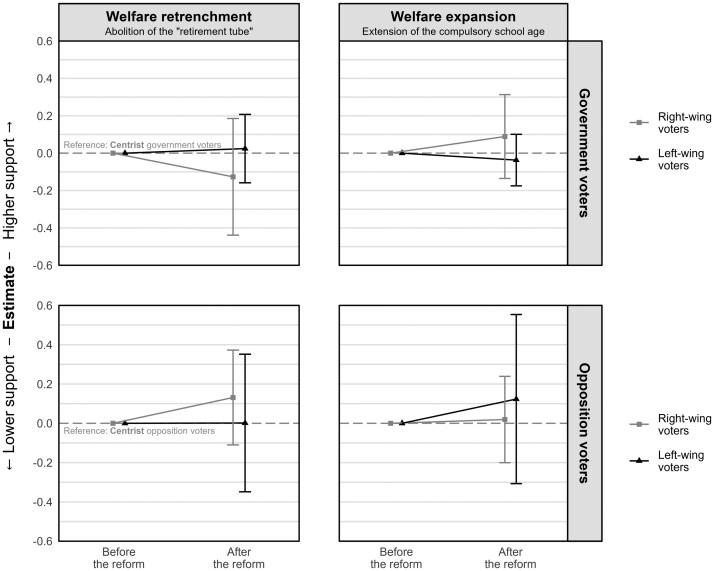
Changes in policy preferences depending on the respondents’ left-right ideological self-placement. Used is the left-right self-placement measured in the first wave using an 11-point scale (0 = left, 10 = right). Responses were recoded into the following groups: 0 to 3 = left wing, 4 to 6 = centrist (reference), and 7 to 10 = right wing. Vertical lines represent 95 percent confidence intervals based on individual cluster-robust standard errors. Coefficients are derived from table F1 in Supplementary Material Section F.

**Figure 4.**
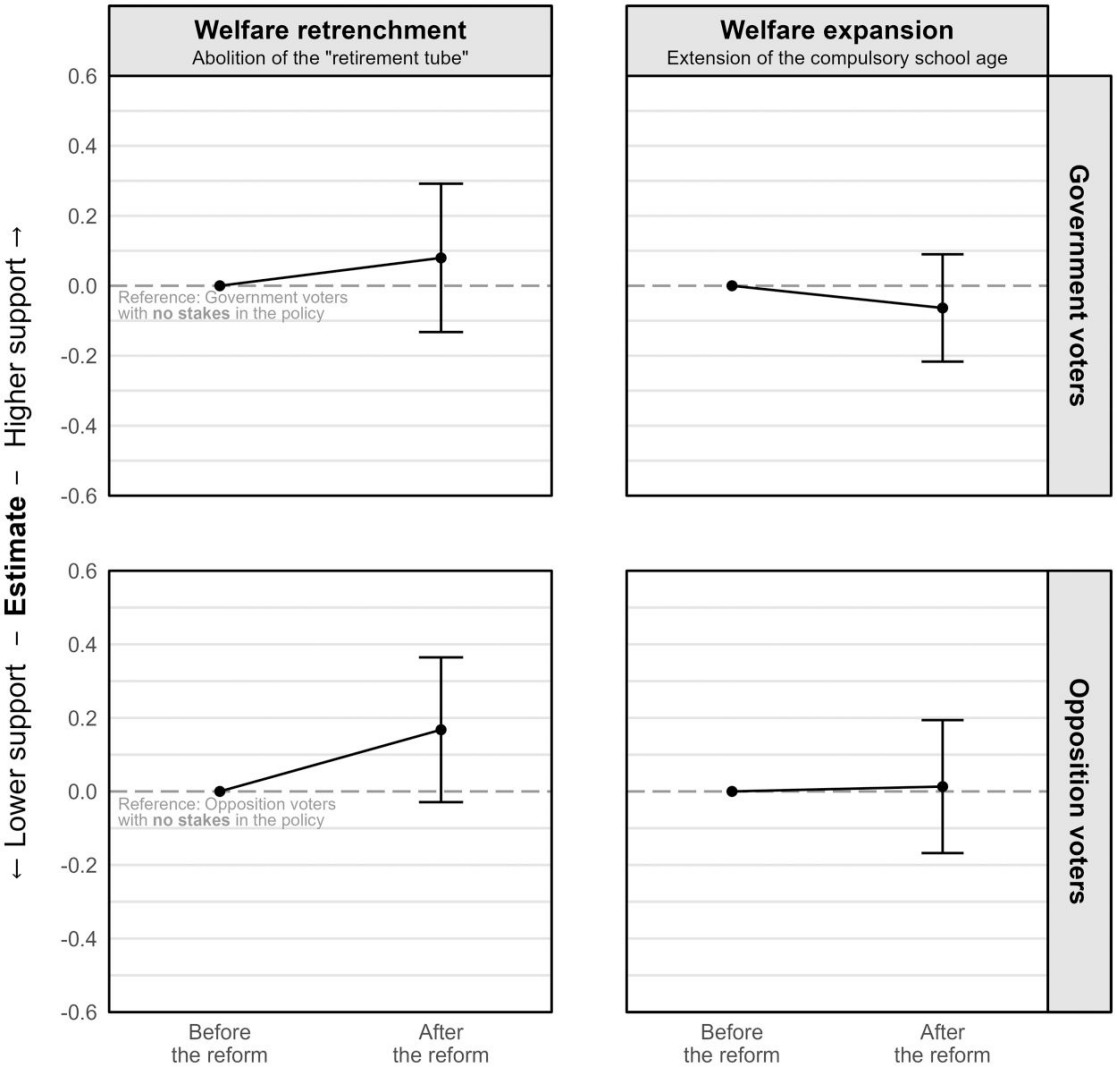
Changes in policy preferences depending on the respondents’ (potential) stake in the policies. Respondents were considered to have a stake in welfare retrenchment if they were employed and aged 55 to 63 (inclusive), and a stake in welfare expansion if they lived in a household with minors. Both measures were collected in the first wave. Vertical lines represent 95 percent confidence intervals based on individual cluster-robust standard errors. Coefficients are derived from table G1 in Supplementary Material Section G.

However, these policies may resonate differently with voters depending on their ideological orientation and the personal self-interest they have invested in the policies. To explore this potential variation, we examine the heterogeneity effect along two dimensions: respondents’ left-right self-placement and their (potential) stake in the policies. Figure 3 illustrates the effect of the policy enactment on both right-wing and left-wing (relative to centrist) government and opposition voters.^7^ We find no significant differences among right-wing and left-wing voters (within government or opposition voters), indicating a consistent response to the policy enactments irrespective of ideological orientation. In figure 4, we further analyze the heterogeneity effect based on an individual’s stake in the policies, while those actively employed and aged between 55 and 63 are considered to have a stake in the welfare retrenchment reform, and households with minors are considered to have a stake in the education reform. Despite those distinctions, we observed no substantial differences in the effects across the two groups.

Moreover, when examining the 95 percent confidence intervals depicted in both figures, it is evident that the null effects cannot be solely attributed to a lack of statistical power. Even with a considerable increase in sample size, pushing the estimated effects beyond any conventional threshold of statistical significance would be challenging.

## Conclusions

We have investigated why voters who generally support the welfare state often seem to overlook or even endorse retrenchment reforms implemented by political parties. Analyzing the Finnish context, where a center-left government initiated both welfare state retrenchment and expansion reforms simultaneously, we have shown that center-left government voters were willing to adjust their policy preferences, even if it meant supporting policies inconsistent with their ideological stances and material stakes in the policies. The findings challenge the notion that voters’ preferences regarding welfare policies are stable and primarily driven by self-interested considerations and deeply embedded values. Instead, our results suggest that partisan cues play a strong role. The employment of a natural quasi-experimental design in a real-world setup enhances the reliability and validity of these findings, which provide a reliable depiction of the partisan influence over public opinion. However, our research design does include some limitations, such as an inability to assess assumptions about parallel trends due to the availability of only two survey waves, and an inability to directly assess voting behavior in the subsequent elections. Altogether, our findings contribute to existing knowledge by highlighting voters’ elasticity in an era of economic scarcity and pressure on governments to implement welfare retrenchment reforms.

## Supplementary Material

nfaf017_Supplementary_Data

## Data Availability

Replication data and documentation are available at https://doi.org/10.7910/DVN/WSZJR9.

## Footnotes

1 The ideological orientation of Finnish voters and their policy preferences are shown in Supplementary Material section D.

2 Only 298 respondents changed their party preference or did not complete the questionnaire in the second wave. A few of the respondents shifted between government and opposition parties: 30 to the opposition parties and 15 to the government parties.

3 See Supplementary Material section A for all variable operationalizations relevant to this research.

4 The number of invitations varies across age groups. Younger panel members are underrepresented and less responsive, so they receive more invitations compared to their older counterparts.

5
Supplementary Material section E shows the distribution of individual-level changes in policy preferences between the two panel waves. It demonstrates that there is enough variance to be explained by exogenous factors such as policy enactment.

6 See Supplementary Material section H for modeling strategy and regression equations.

7 See Supplementary Material section D for the distribution of government, opposition, and individual party electorates along the left-right dimension.
