# Supplementary material for: Partisan Influence on Policy Preferences in Retrenching the Welfare State
Source: Public Opin Q. 2025 May 29;89(2):424–44. doi: 10.1093/poq/nfaf017 (PMC12369940; doi:10.1093/poq/nfaf017)
Supplement: nfaf017_Supplementary_Data [file nfaf017_supplementary_data.pdf]

Supplementary Material for

Partisan Influence on Policy Preferences in Retrenching the Welfare State

Miroslav Nemčok *
University of Oslo
miroslav.nemcok@stv.uio.no

Hanna Wass
University of Helsinki
hanna.wass@helsinki.fi

Juho Vesa
University of Helsinki
juho.vesa@helsinki.fi

Correspondence to: miroslav.nemcok@stv.uio.no

This file includes:

A Variable operationalizations 2
B Comparison of policy preferences among electorates of individual parties 5
C Descriptive statistics: Opposition party voters 9
D Ideological profiles of the electorates 10
E Distribution of within-individual opinion shifts 13
F Effect heterogeneity: Ideological self-placements 14
G Effect heterogeneity: Having stakes in the policies 15
H Regression equations 16

A Variable operationalizations

POLICY PREFERENCE	
**Wave 1**	September 1–3, 2020
The COVID-19 pandemic has driven societies into a period of crisis and made it more difficult to find solutions to many already prevailing social problems. In this survey, we study citizens' support for different policy solutions aimed at enhancing the economy and employment.<br><br>What do you think about the following policy proposals?	
Abolish the "retirement tube," which allows elderly individuals who are unemployed to receive income-based unemployment compensation until reaching retirement age.	
Unemployment benefits will be structured in tiers, initially providing a larger benefit than currently, but gradually decreasing as unemployment persists, and being paid for a shorter duration.	
Changes will be made to unemployment benefits so that the earnings-related daily allowance will no longer contribute to pension benefits in the future.	
The system of partial unemployment benefits, which provides compensation for reduced working hours, will be phased out.	
Wage increases will be implemented at a slower rate compared to competitor countries, making it more advantageous for employers to hire workers.	
Working hours will be extended or adjusted to reduce overtime costs for employers.	
The practice of negotiating terms of employment and wages locally within companies will be expanded.	
A transition to a six-hour workday will be pursued.	
Adult education support will be discontinued, leaving study grants or the option to study with unemployment benefits as the primary means of support for adult education, especially for courses that promote employment.	
Monthly credit requirements for study points will be strengthened to accelerate progress in studies.	
Extend the compulsory schooling age to 18 years.	
Ensure that secondary education is fully free of charge, covering not only tuition but also study materials and tools.	

[1] strongly disagree	[2] disagree	[3] agree	[4] strongly agree	Don't know

**Wave 2**				February 12–17, 2021
The COVID-19 pandemic has driven societies into a period of crisis and made it more difficult to find solutions to many already prevailing social problems. In this survey, we study citizens' support for different policy solutions aimed at enhancing the economy and employment.<br><br>The Finnish Parliament has recently approved the following reforms. What do you think about this decision?				
Abolish the "retirement tube," which allows elderly individuals who are unemployed to receive income-based unemployment compensation until reaching retirement age.				
Extend the compulsory schooling age to 18 years.				
Ensure that secondary education is fully free of charge, covering not only tuition but also study materials and tools.				
[1] strongly disagree	[2] disagree	[3] agree	[4] strongly agree	Don't know

**PARTY VOTE**	
**Wave 1 and 2**	September 1–3, 2020 and February 12–17, 2021
If the parliamentary elections were held now, which party would you vote for?	
Social Democrats	Government parties
Centre Party	
Left Alliance	
Greens	
Swedish People's Party	
National Coalition	Opposition parties
Finns Party	
Christian Democrats	
Movement Now	
other	

**POLITICAL IDEOLOGY**			
**Wave 1 and 2**			September 1–3, 2020 and February 12–17, 2021
In politics, people sometimes talk about the left and the right. Where would you place yourself on a scale of zero to ten when zero means left and ten right?			
[0] Left	…	[10] Right	Don't know

HAVING STAKES IN POLICIES: WELFARE RETRENCHMENT	
**Wave 1**	September 1–3, 2020
Age (numeric)	Coded as
If the numeric age is greater than or equal to 55 AND less than or equal to 63	Stakes in the policy
Age below 55 or above 63	No stakes in the policy

Occupation	Coded as
[1] Self-employed	No stakes in the policy
[2] Executive position	Stakes in the policy
[3] Upper non-manual	Stakes in the policy
[4] Lower non-manual	Stakes in the policy
[5] Manual	Stakes in the policy
[6] self-employed in agricultural sector	No stakes in the policy
[7] student	No stakes in the policy
[8] pensioner	No stakes in the policy
[9] staying home with children	No stakes in the policy
[10] unemployed	No stakes in the policy
Don’t know	No stakes in the policy

Note: Both conditions — *age* and *occupation* — must be met for a person to be categorized as having stakes in the welfare retrenchment.

HAVING STAKES IN POLICIES: WELFARE EXPANSION	
**Wave 1**	September 1–3, 2020
Household composition	Coded as
[1] Single	No stakes in the policy
[2] Couple without children	No stakes in the policy
[3] Other household with only adults	No stakes in the policy
[4] Household with minors	Stakes in the policy
Don’t know	No stakes in the policy

B Comparison of policy preferences among electorates of individual parties

Figure 2 for voters of the government parties, presented in the main manuscript, and Figure C1 for voters of the opposition parties, presented below, provide an overview of changes in policy preferences before and after the reform enactments among individual party electorates. However, these figures dichotomized the original four-point scale measuring voters' policy preferences as *strongly disagree*, *disagree*, *agree*, *strongly agree* (and *don't know*).

Therefore, this section includes a detailed breakdown of measured policy preferences using all four ordinal categories and their distribution among individual electorates of government parties (Figure B1, first part on p. A6), individual electorates of opposition parties (Figure B1, second part on p. A7), as well as aggregated policy preferences for all government and opposition parties as a whole. These figures support the claim made in the section introducing the empirical context, "The empirical context: two structural welfare reforms in Finland," included in the main manuscript: "Their [i.e., the reforms'] initial popularity differed, with the removal of the 'retirement tube' being considerably less popular than the extension of compulsory schooling."

Figure B1. Government supporters: Changes in policy preferences among voters of he individual government parties in the two rounds of the survey.

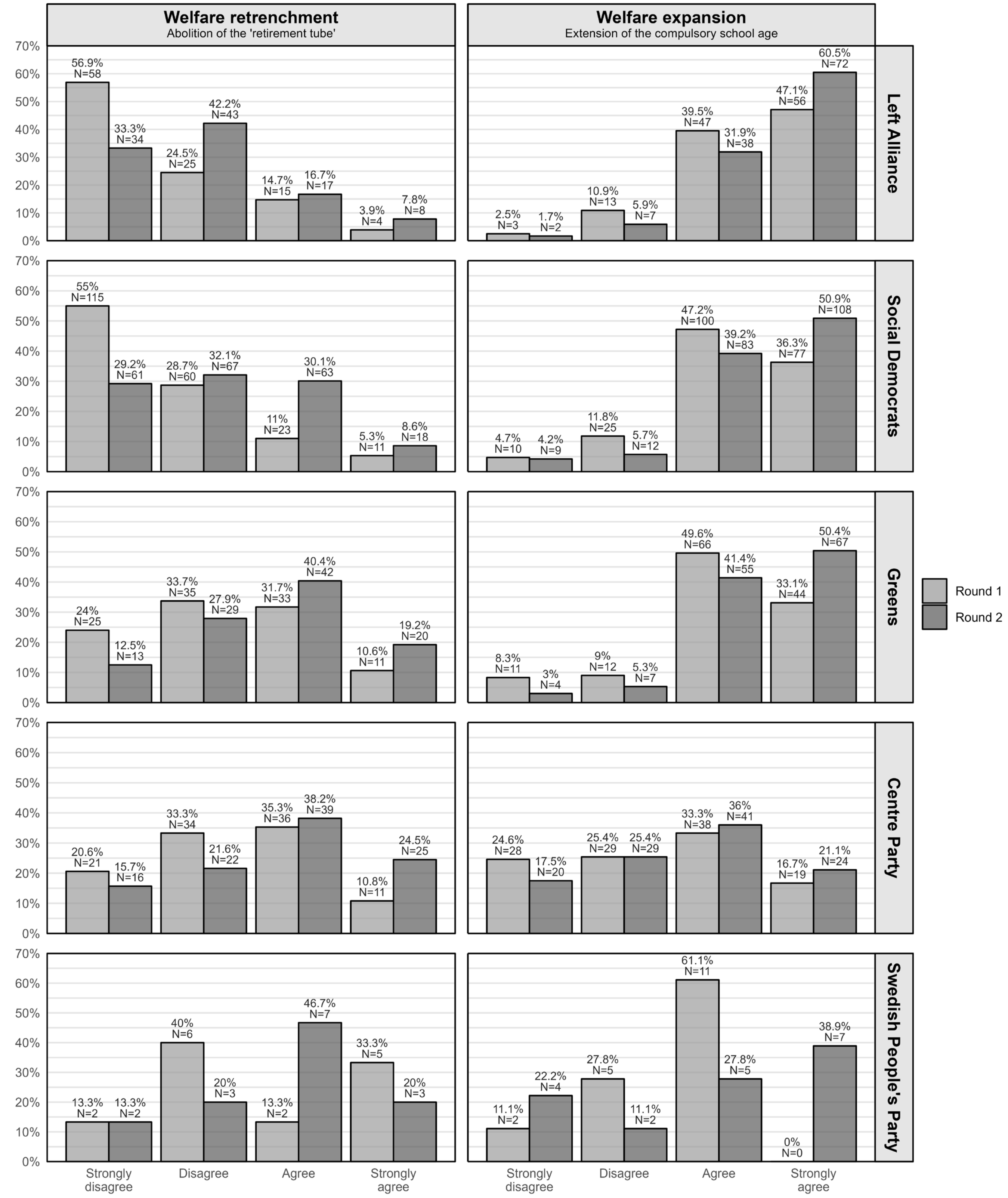

Figure B1. (*cont.*) Opposition supporters: Changes in policy preferences among voters of the individual opposition parties in the two rounds of the survey.

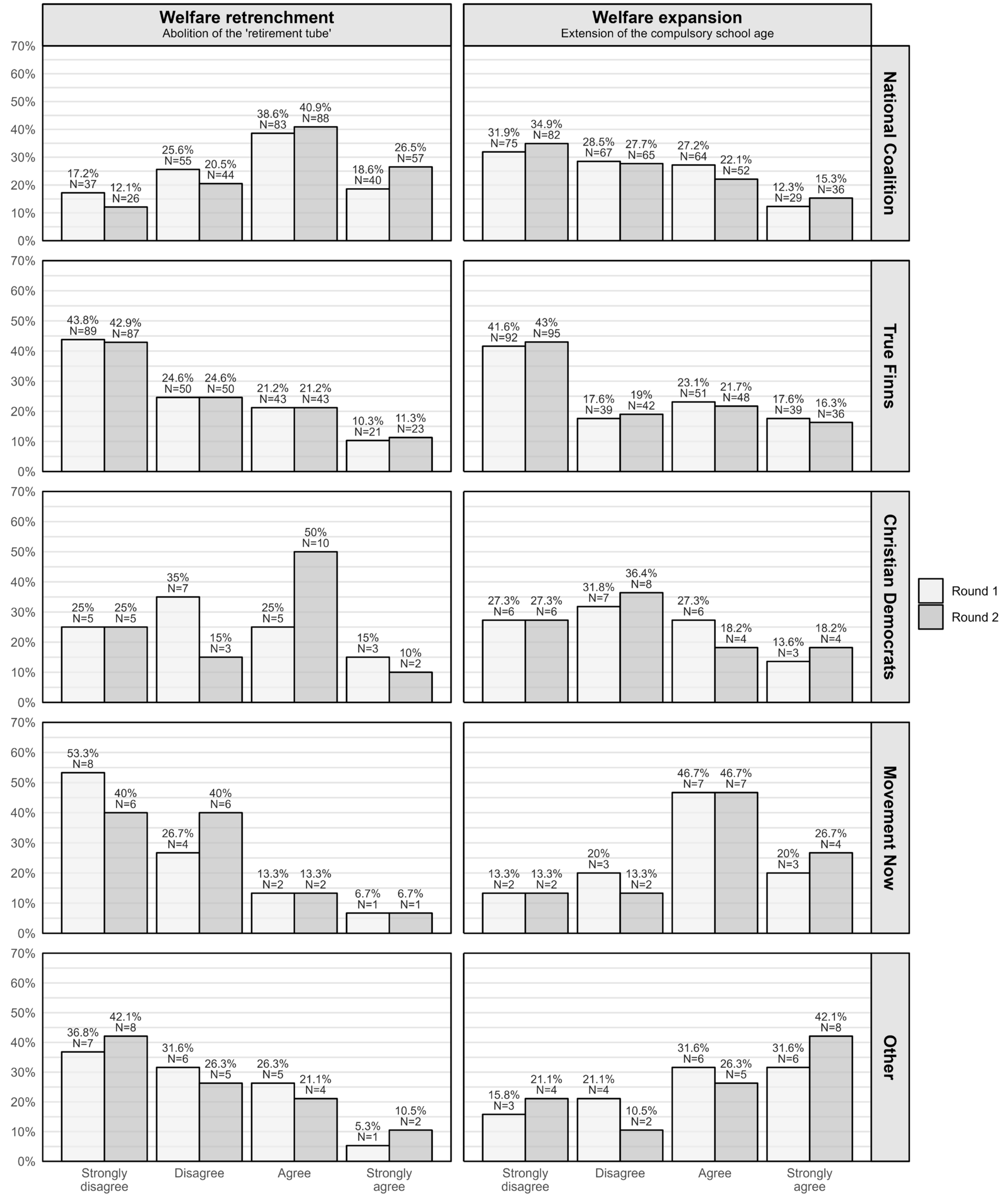

Figure B2. Changes in policy preferences among government and opposition voters in two rounds.

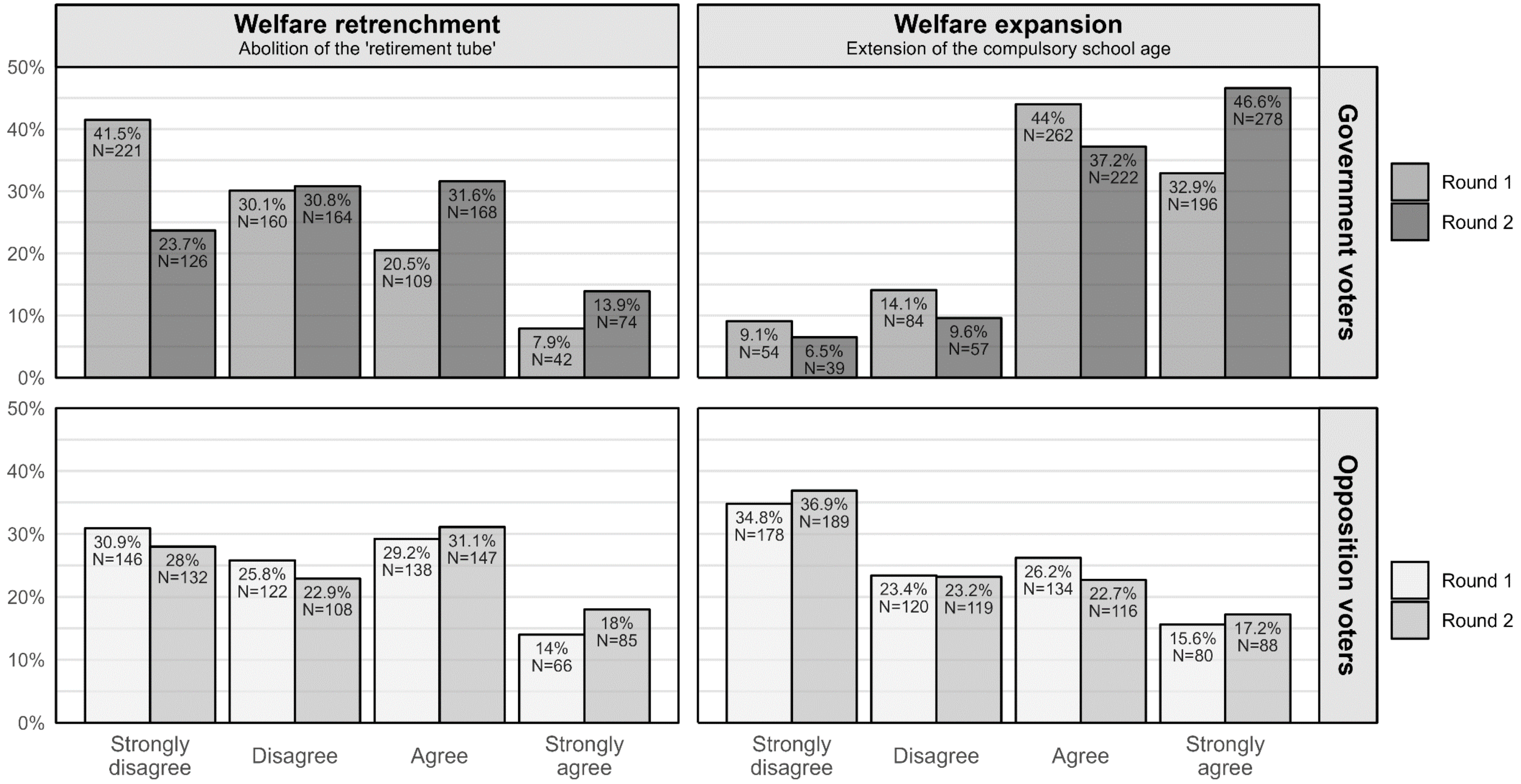

C Descriptive statistics: Opposition party voters

Figure C1. Descriptive statistics: Left-right self-positioning of *opposition* party voters and changes in their policy preferences. Dashed line represents the average.

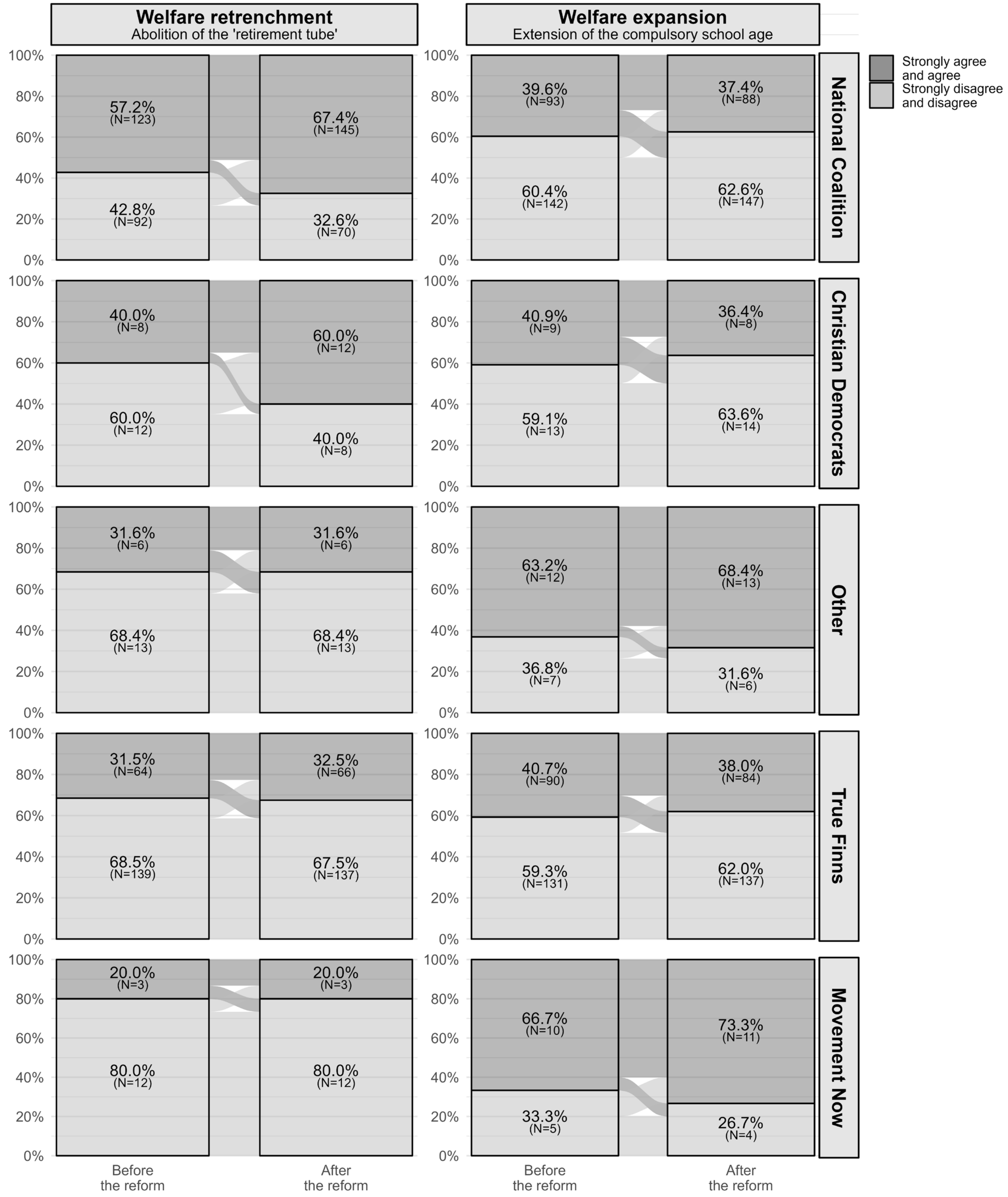

D Ideological profiles of the electorates

To assess whether government and opposition voters in Finland position themselves distinctly on the left-right scale, the survey asked respondents the following: “In politics, people sometimes talk about the left and the right. Where would you place yourself on a scale of zero to ten, when zero means left and ten right?” As the wording implies, the question was administered on an 11-point scale with two anchors, 0=left and 10=right. Figure D1 compares the distribution between government vs. opposition electorates, while Figure D2 further breaks down this distribution to the individual parties.

Figure D1 shows the kernel densities for both groups, strongly confirming that the political orientations of the two groups appear as if they were drawn from two different populations. Moreover, as illustrated in the lower part of the figure, their ideological placement remained similar in the second round of data collection. This demonstrates that the government voters positioned themselves on the left side consistent with the center-left ideology attributed to the Finnish government parties during the period relevant for this research. Opposition voters were prone to place themselves on the right.

Figure D1. Kernel density plots: Distribution of government versus opposition voters on the left-right dimension; dashed lines indicate group averages.

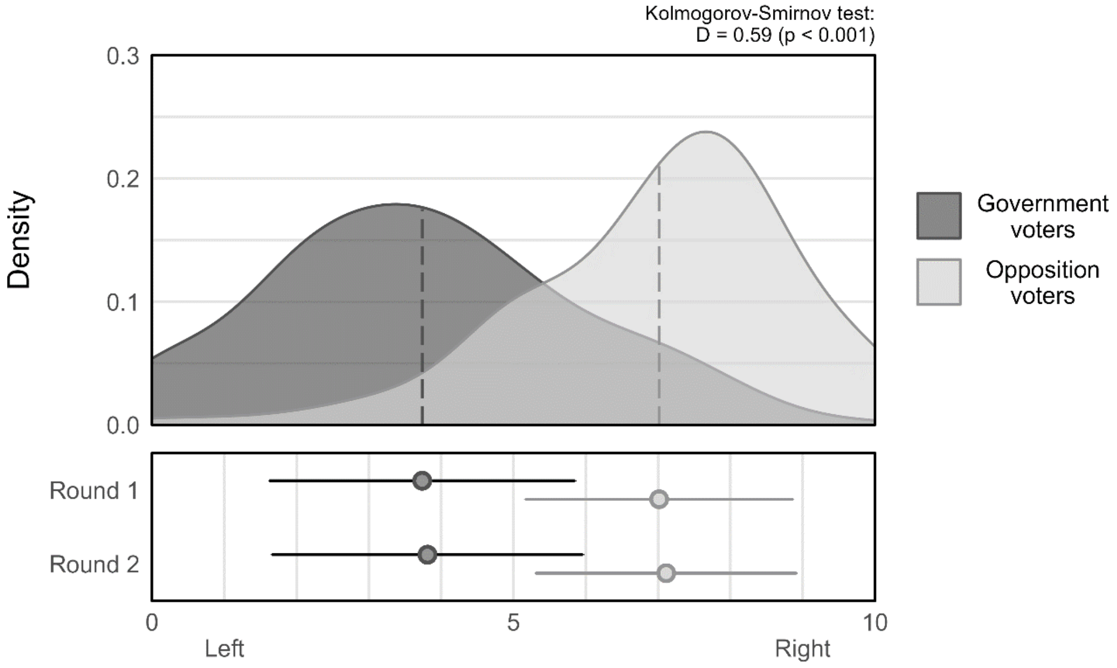

Figure D2. Kernel density plots: Comparison of voters' left-right ideological self-placement across the parties in Finland; dashed lines indicate group averages.

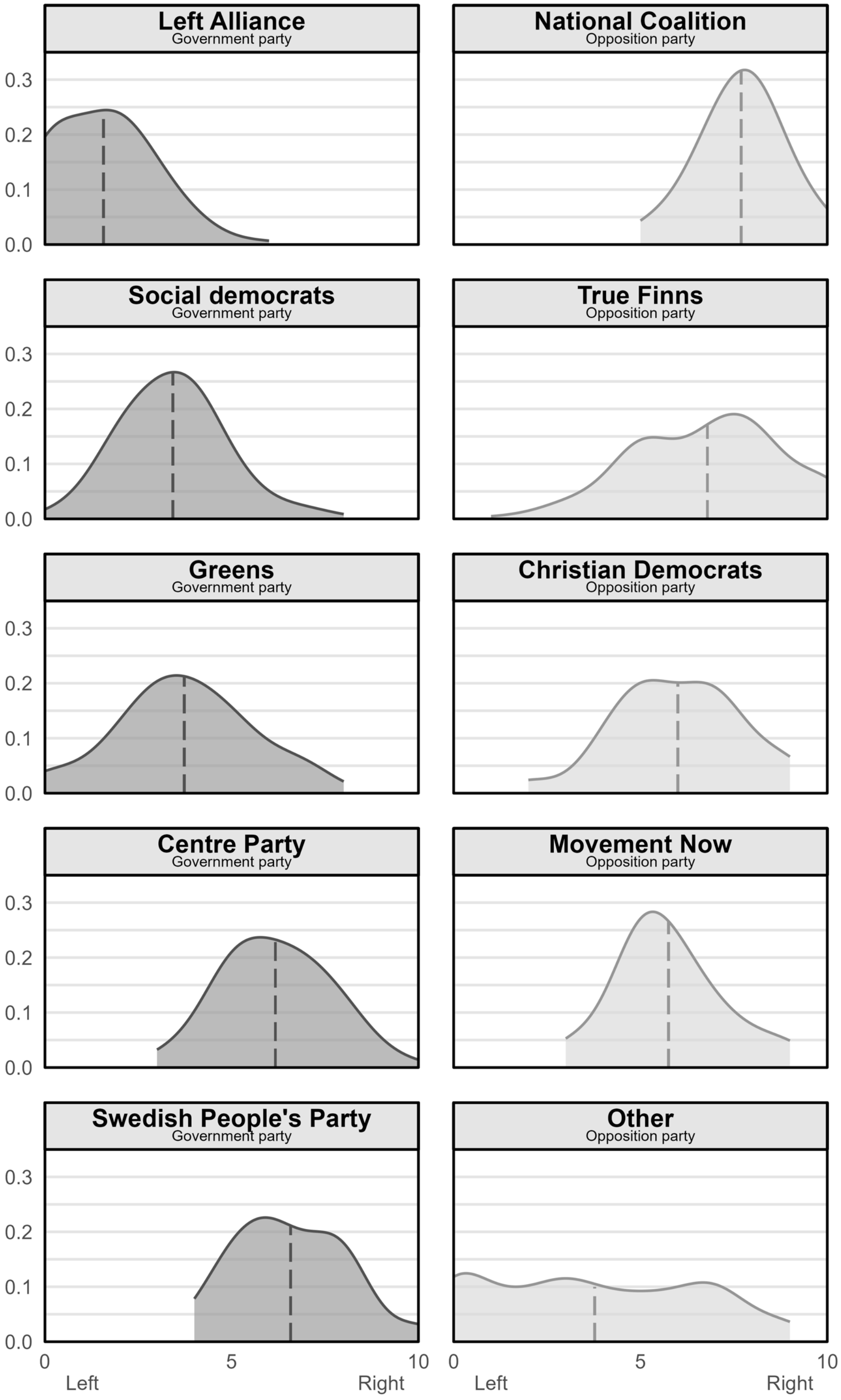

E Distribution of within-individual opinion shifts

Respondents who participated in both waves of our panel survey were repeatedly asked about their opinions regarding the two policy reforms: the abolition of the "retirement tube" and the extension of the compulsory schooling age. Hence, we were able to calculate the opinion shifts of the same individuals in the two policy domains. The histograms in Figure E1 demonstrate that the data include sufficient variance over time, which could be attempted to be explained by the presented analysis.

Figure E1. Histograms: Distribution of within-individual opinion shifts between the two panel waves.

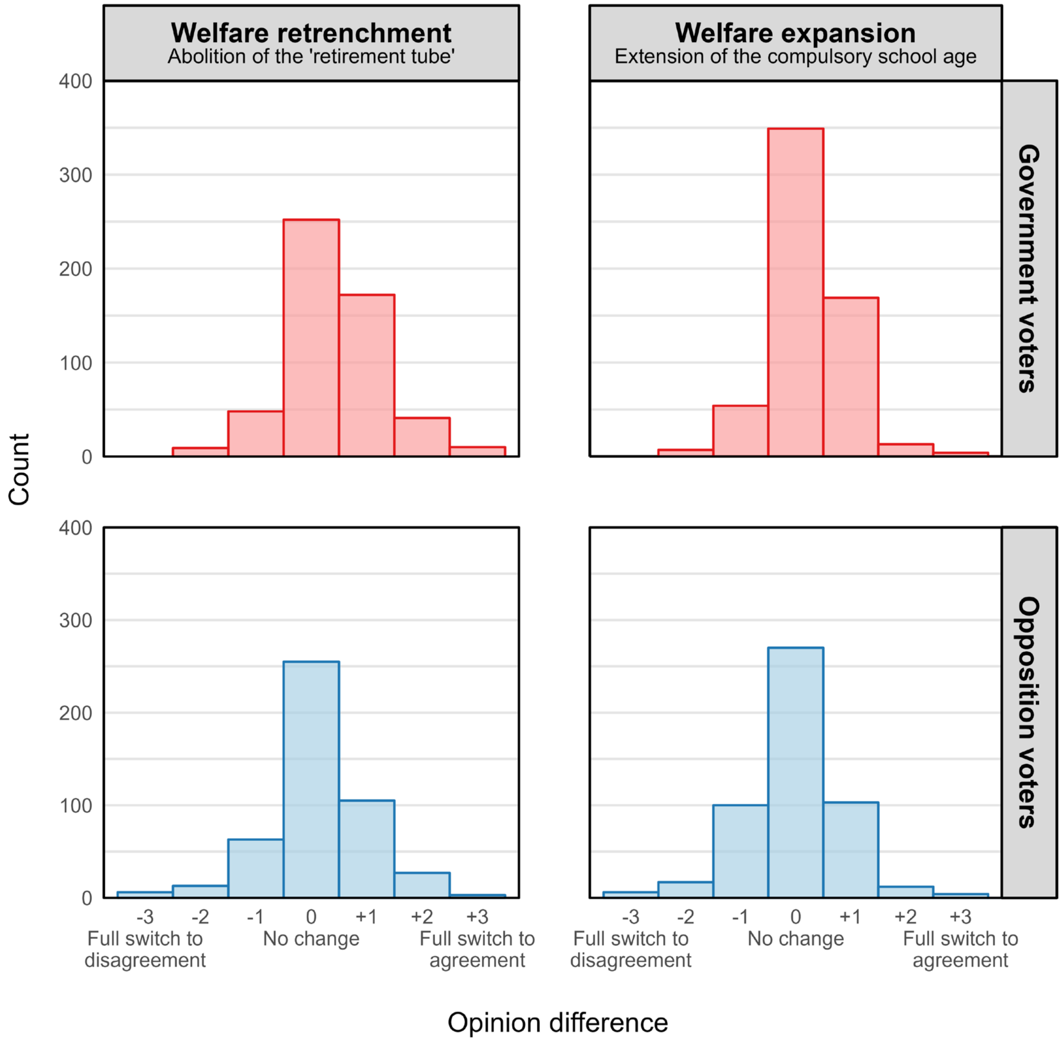

F Effect heterogeneity: Ideological self-placements

Table F1. Effect heterogeneity: Regression analysis of the shifts in policy preferences among government and opposition voters depending on their left-right ideological self-placement in the first wave.

	**Welfare retrenchment:** Abolition of the 'retirement tube'						**Welfare expansion:** Extension of the compulsory schooling age					
	(F1)			(F2)			(F3)			(F4)		
Subset:	Government voters			Opposition voters			Government voters			Opposition voters		
After the reform * Centrist voters	Ref.			Ref.			Ref.			Ref.		
After the reform * Left-wing voters	0.024	[0.093]	(0.794)	0.002	[0.178]	(0.993)	-0.037	[0.070]	(0.598)	0.123	[0.219]	(0.574)
After the reform * Right-wing voters	-0.127	[0.159]	(0.426)	0.131	[0.123]	(0.286)	0.089	[0.114]	(0.436)	0.019	[0.112]	(0.864)
Fixed-Effects:												
Individuals			Yes			Yes			Yes			Yes
Left-right group			Yes			Yes			Yes			Yes
Round			Yes			Yes			Yes			Yes
S.E.: Clustered			by individuals			by individuals			by individuals			by individuals
Observations			1,064			944			1,192			1,024
R^2			0.83815			0.83990			0.87481			0.84910
Within R^2			0.00279			0.00372			0.00308			0.00058

Note: Coefficients are predicted values based on OLS regression with individual cluster-robust standard errors in brackets and p-values in parentheses.

G Effect heterogeneity: Having stakes in the policies

Table G2. Effect heterogeneity: Regression analysis of the shifts in policy preferences among government and opposition voters depending on their (potential) stakes in the enacted policies based on the information from the first wave.

	**Welfare retrenchment:** Abolition of the 'retirement tube'						**Welfare expansion:** Extension of the compulsory schooling age					
	(G1)			(G2)			(G3)			(G4)		
Subset:	Government voters			Opposition voters			Government voters			Opposition voters		
After the reform * No stakes		Ref.			Ref.			Ref.			Ref.	
After the reform * Stakes in the policy	0.080	[0.108]	(0.460)	0.168	[0.100]	(0.095)	-0.064	[0.078]	(0.417)	0.013	[0.092]	(0.886)
Fixed-Effects:												
Individuals			Yes			Yes			Yes			Yes
Stakes in the policy group			Yes			Yes			Yes			Yes
Round			Yes			Yes			Yes			Yes
S.E.: Clustered			by individuals			by individuals			by individuals			by individuals
Observations			1,064			944			1,192			1,024
R^2			0.80238			0.81246			0.84196			0.83551
Within R^2			0.00093			0.00335			0.00114			0.00004

Note: Coefficients are predicted values based on OLS regression with individual cluster-robust standard errors in brackets and p-values in parentheses.

H Regression equations

Table 2 includes a set of regression models estimating the change in the policy preference gap between government and opposition voters over time. The estimated models are two-way fixed effects models using the first-difference estimator. The corresponding regression equation is as follows:

$$\Delta PolicyPref_{it} = \beta_1 \Delta Time_{it} + \beta_2 (\Delta Time_{it} \times PartyPref_i) + \beta_3 \Delta Ideology_{it} + \epsilon_{it}$$

Where: $\Delta PolicyPref_{it}$ represents the first difference of the dependent variable, i.e., policy stances towards welfare retrenchment or welfare expansion policy, for individual i between time periods t and $t-1$. $Time_{it}$ is the time-fixed effect for time period t, i.e., whether the observation was collected in the first or second wave of the panel survey. $PartyPref_i$ is the individual-specific dummy for either government or opposition supporting individual i at time t. Thus, $\Delta Time_{it}$ captures the first difference for individual i between time periods t and $t-1$. Since the $PartyPref_i$ dummy is coded as 0 for the opposition supporters, β_1 represents the first difference for opposition voter i between time periods t and $t-1$. β_2 represents the first difference for government voter i between time periods t and $t-1$, on top of the first difference estimated for opposition voters as β_1. Since the regression includes only those individuals whose party preference remained consistent across both waves, the specified model does not estimate any effect over time for a change in $PartyPref_i$.

$\Delta Ideology_{it}$ is the independent variable represented by a self-placement on the left-right scale of individual i at time t. It aims to control for possible time-variant movements along the left-right dimension in models 2 and 4. Finally, ϵ_{it} is the error term.

Figures 3 and 4 extend the analysis to address potential effect heterogeneity depending on individuals' political ideology (Figure 3 based on table F1 in section F of the online appendix), and material stakes in the enacted policies (Figure 4 based on table G1 in section G of the online appendix). The corresponding regression equation for models F1 to F4 is as follows:

$$\Delta PolicyPref_{it} = \beta_1(\Delta Time_{it} \times PartyPref_i \times Ideology_i) + \epsilon_{it}$$

in which $Ideology_i$ is represented by a dummy measured in the first survey wave on an 11-point scale (0=left, 10=right). Individuals' responses were recoded into three groups as follows: 0 to 3 = left wing, 4 to 6 = centrist (reference), and 7 to 10 = right wing, which is why Table F1 provides predicted values for each group.

The corresponding regression equation for models G1 to G4 is as follows:

$$\Delta PolicyPref_{it} = \beta_1(\Delta Time_{it} \times PartyPref_i \times Stake_i) + \epsilon_{it}$$

in which $Stake_i$ is a binary dummy coded as 1 if individual i is considered to have stake in the policies. Individuals were deemed to have a vested interest in welfare retrenchment if they were actively employed and aged between 55 and 63 (inclusive). Additionally, individuals were regarded as having a stake in welfare expansion if they resided in a household with minors. All relevant measures were included in the first wave, or among the socio-economic background

information monitored by the hired survey agency *Taloustutkimus* while facilitating their online panel.
